# Perichondritis: Not All Ear Pain Is Otitis

**DOI:** 10.7759/cureus.11141

**Published:** 2020-10-24

**Authors:** Mark D Rivera-Morales, Jennifer L Rodríguez-Belén, Ariel Vera, Latha Ganti

**Affiliations:** 1 Emergency Medicine, University of Central Florida College of Medicine, Orlando, USA; 2 Emergency Medicine, Osceola Regional Medical Center, Kissimmee, USA; 3 Medicine, Ponce School of Medicine, Ponce, PRI; 4 Emergency Medicine, Envision Physician Services, Nashville, USA; 5 Emergency Medical Services, Polk County Fire Rescue, Bartow, USA

**Keywords:** perichondritis, fluoroquinolones

## Abstract

Acute auricular perichondritis is an infection and inflammatory disease of the external ear that can potentially cause serious complications if not diagnosed and treated promptly. Delays in treatment can lead to devastating focal cartilage necrosis and, subsequently, permanent deformities of the ear. We present the case of a two-year-old boy who was diagnosed with acute perichondritis after presenting to the emergency department (ED) with acute ear redness, swelling, and tenderness. In this article, we will discuss how the diagnosis of perichondritis is made and give a brief literature review on the management approaches and the reasoning behind them. Particularly, we address the dilemma of whether fluoroquinolones use in pediatric patients is safe and warranted in this disease entity, based on the latest evidence.

## Introduction

Auricular perichondritis (also called pinna perichondritis) is an infectious and inflammatory condition of the external ear that usually occurs secondary to trauma (i.e., high ear piercing, blunt trauma, burns, iatrogenic), which leads to the infection, with or without abscess formation [1]. It classically presents as redness and painful swelling of the auricle with sparing of the lobule. It is important for the emergency department (ED) and/or primary care physician to identify early and correctly treat the perichondritis since incorrect or delayed treatment could lead to focal cartilage necrosis and subsequent, permanent deformities of the external ear (“cauliflower ear”). In addition, if an abscess formation is suspected or clinically identified, the patient needs parenteral antibiotics and prompt ears nose throat (ENT) specialist evaluation, as incision and drainage with possible cartilage necrotic tissue debridement may be needed. 

## Case presentation

**Figure 1:**
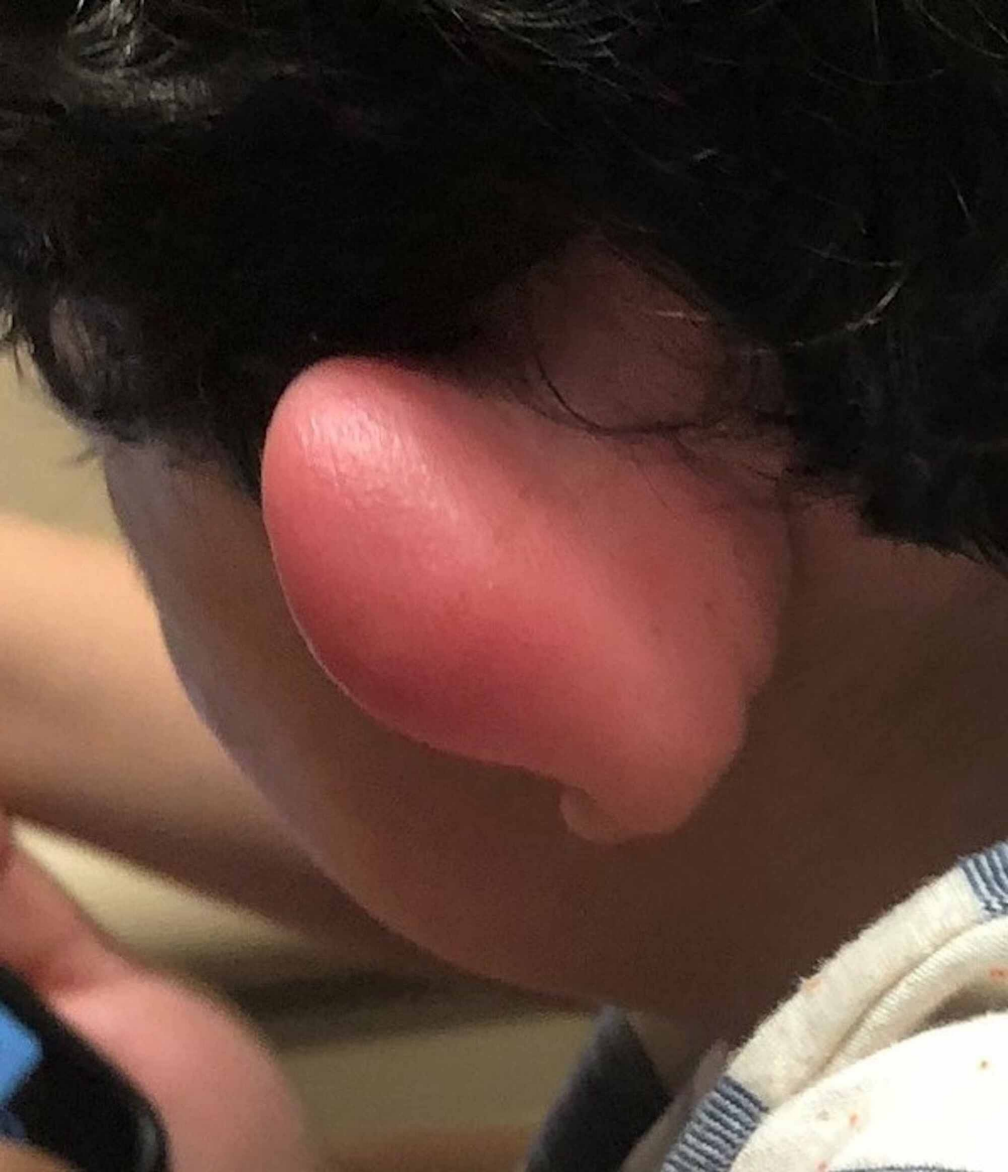
View of the posterior aspect of the pinna demonstrating erythema and edema

**Figure 2:**
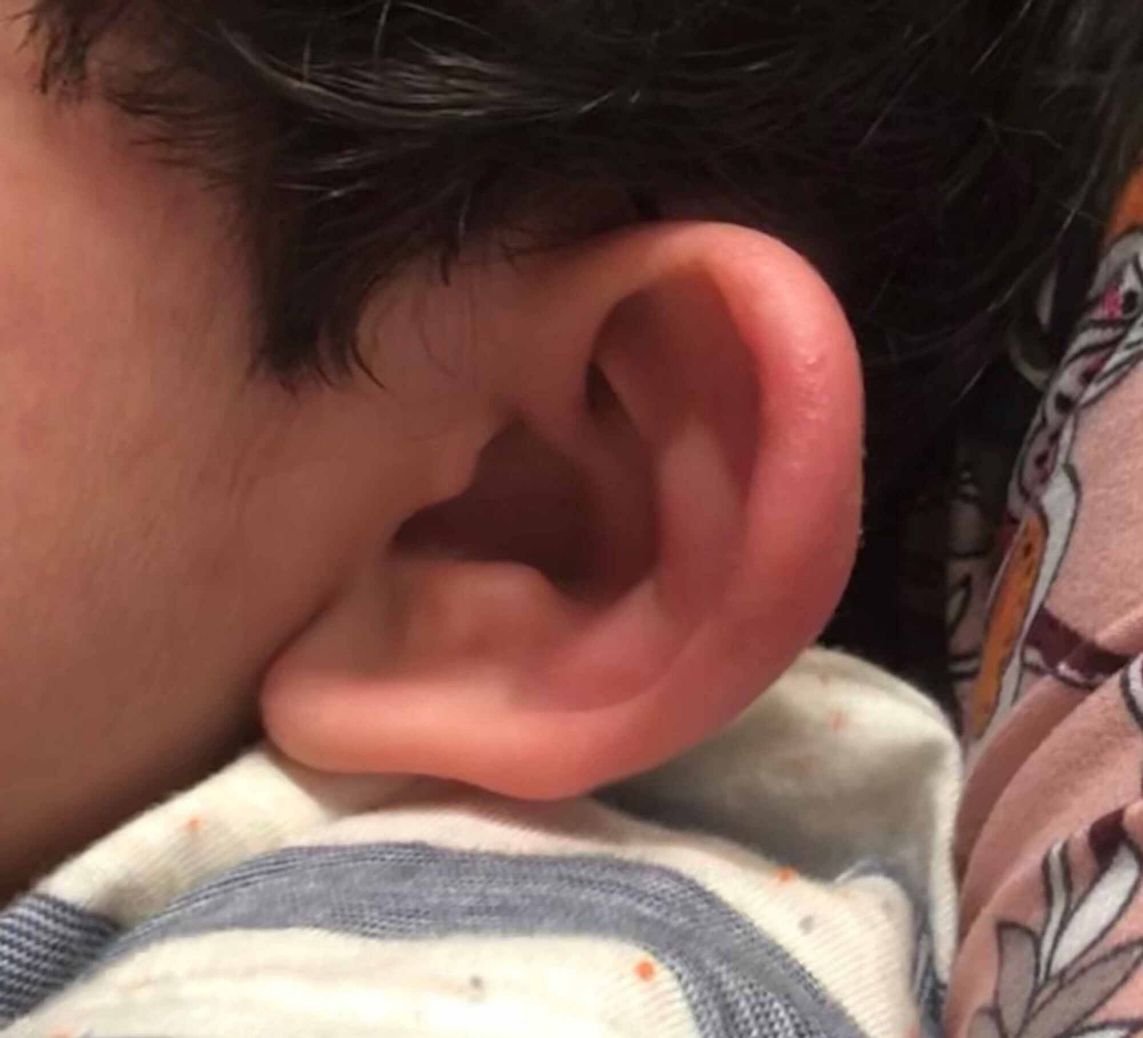
View of the anterior aspect of the pinna demonstrating erythema and edema

A two-year-old boy with no past medical history presented to our pediatric ED brought by his mother due to worsening left ear redness and swelling that started two days prior. The mother believed it started after an insect bite to the auricle of the left ear, causing the patient to frequently scratch the ear. There was no reported fever, otorrhea, impaired hearing, previous history of otitis externa, otitis media, skin infections, or other symptoms. On physical exam, the left ear appeared erythematous and swollen in the helix and antihelix area (Figure 1), with swelling best noted on the posterior aspect of the auricle (Figure 2).

On palpation, there was tenderness of the auricle, warmth and fluctuant swelling in the affected area. There was sparing of the earlobe and tragus. The mastoid area was not tender or swollen. The ear canal was clear without evidence of otitis externa, and the tympanic membrane was normal. The remainder of the patient’s physical exam, including vital signs, was normal.

The diagnosis was made by history and physical exam findings. Important features that help distinguish this case from an expanding otitis externa or otitis media were the sparing of the ear lobule and normal ear canal and tympanic membranes. Other differential diagnoses considered included malignant otitis externa, mastoiditis, and auricular hematoma, but each of these has distinctive features that are not present in perichondritis and are well described in the literature, therefore will not be discussed in this case report. 

The patient was treated with ibuprofen 10 mg/kg, one dose of intravenous (IV) piperacillin/tazobactam 100 mg/kg (piperacillin component), and a single dose of dexamethasone 4 mg IV. After discussion via phone call with the hospitalist and ENT specialist, we decided to admit the patient to the pediatric inpatient unit for close monitoring and evaluation by ENT during the next 24 hours. The discussion of whether to use a fluoroquinolone to cover *Pseudomonas *came up, but it was decided to continue IV piperacillin/tazobactam 100 mg/kg every eight hours, based on local antibiogram guidance. 

The patient was evaluated by ENT the next day and was found to have significantly improved. He did not have any fluctuance, which ruled out the initial suspicion of a developing abscess or suppurative perichondritis. The patient was discharged home at the end of admission day one, after discussing with the patient's mother the need to have outpatient follow-up within that same week and to continue giving oral antibiotics at home as prescribed by the ENT specialist. The patient received a total of four doses of IV piperacillin/tazobactam 100 mg/kg during admission and was discharged with a prescription for oral cefdinir 7 mg/kg every 12 hours for 10 days. The patient’s mother was contacted via phone call and reported that the patient’s symptoms resolved completely on approximately day four of treatment and the patient completed a full 10-day course of antibiotic. She did not attend the follow-up visit with ENT or pediatrician as the patient was feeling well. On a three-month post-ED visit phone call, the mother denied perichondritis recurrence and reported no other complications or cosmetic sequelae.

## Discussion

The organism most commonly isolated in suppurative (abscess) perichondritis is *Pseudomonas aeruginosa*. Other organisms commonly found include *Staphylococcus aureus* or *Escherichia coli* and *Proteus *species [1,2]. Meanwhile, in non-abscess perichondritis, *S. aureus* is the most commonly isolated organism, followed by *P. aeruginosa* [2]. A retrospective study of 114 patients admitted for perichondritis concluded that empirical treatment should include parenteral antipseudomonal agents [3]. This approach of treating empirically for *P. aeruginosa* in all cases of perichondritis is described in multiple case series and retrospective studies published in literature [1,3,4]. A different approach was suggested in a recent retrospective study of 112 patients in which the authors concluded that in non-abscess perichondritis, empirical treatment with antibiotics covering *S. aureus *(instead of using antipseudomonal agents, as is the case for suppurative perichondritis) is adequate [2]. Unfortunately, there is currently no meta-analysis or randomized controlled trial that could guide and further allow us to reach a conclusion on which of the approaches mentioned above are more effective.

However, outpatient treatment with fluoroquinolones can still be a challenge to physicians, not because of the adverse effects but because of the increasing antibiotic resistance patterns [4]. These patterns vary widely, and it could be applied to other antimicrobials as well, not only fluoroquinolones. We recommend considering the local antibiograms when deciding whether to treat outpatient or inpatient and choosing the correct empiric antibiotic. Possibly related to this risk of treatment failure, some authors routinely recommend hospital admission for all cases of perichondritis, as patients will benefit from urgent specialist evaluation and parenteral antibiotic therapy, particularly among pediatric patients [5].

Fluoroquinolones, such as ciprofloxacin, have excellent tissue penetration and maybe the only true oral anti-pseudomonal drugs available. Unfortunately, in pediatrics, the use of both systemic and topical fluoroquinolones has been historically restricted due to the apparent risks of damage to growing cartilage and arthropathies [6,7]. This limitation of fluoroquinolone use in pediatrics has been controversial, and the adverse effects are possibly overestimated by small case series or single case reports and anecdotal evidence.

Adefurin et al. studied the safety profile of ciprofloxacin in a meta-analysis of 105 articles and a total of 16184 patients in which ciprofloxacin was used in any pediatric age group (less than 17 years old) [6]. A total of 1065 adverse events (AEs) were reported, which would give an estimated risk of 7%, or one AE in 14 pediatric patients taking ciprofloxacin. The most common AE was musculoskeletal (estimated risk of 1.6%, or one in 62.5 patients), followed by abnormal liver function tests, nausea, changes in white blood cell count, and vomiting. Of the reported musculoskeletal AEs, 50% were arthralgias (mostly of the knee joint), 19% tendon disorder, and 15% were reduced movement/stiffness. The dose of ciprofloxacin used in most of the studies considered in the metanalysis ranged between 10-30 mg/kg/day divided into two daily doses, with a median duration of use of 14 days.

The American Academy of Pediatrics (AAP) also has addressed this controversy several times, with the latest report published in 2016, in which they concluded that “No compelling published evidence to date supports the occurrence of sustained injury to developing bones or joints in children treated with available fluoroquinolone agents.” Nonetheless, the FDA safety data of ciprofloxacin still includes the suggestion for musculoskeletal adverse events. Of note, there was no specific discussion or recommendations about systemic or topical fluoroquinolones use in infants younger than six months of age in the AAP report mentioned above [7].

## Conclusions

Acute auricular perichondritis should be diagnosed promptly by the ED or primary care physician, as delayed diagnosis and treatment may lead to liquefactive cartilage necrosis and permanent cosmetic outcomes. If there is an abscess or fluctuant swelling, management warrants hospital admission for parenteral anti-pseudomonal agents and evaluation by an ENT specialist for possible incision and drainage with tissue debridement if needed. If attempting initial outpatient management, oral fluoroquinolones could be used safely in a pediatric patient, as long as there is adequate close outpatient follow-up for treatment response and monitoring for adverse events.
